# Elderly Perception on the Internet of Things-Based Integrated Smart-Home System

**DOI:** 10.3390/s21041284

**Published:** 2021-02-11

**Authors:** Tae Hee Jo, Jae Hoon Ma, Seung Hyun Cha

**Affiliations:** 1 Department of Computer Science & Engineering, Hanyang University, Seoul 04763, Korea; thjo94@hanyang.ac.kr; 2 Department of Interior Architecture Design, Hanyang University, Seoul 04763, Korea; jaehunvv@hanyang.ac.kr

**Keywords:** smart home, aging-in-place, perception, IoT sensors, elderly

## Abstract

An integrated smart home system (ISHS) is an effective way to improve the quality of life of the elderly. The elderly’s willingness is essential to adopt an ISHS; to the best of our knowledge, no study has investigated the elderly’s perception of ISHS. Consequently, this study aims to investigate the elderly’s perception of the ISHS by comprehensively evaluating its possible benefits and negative responses. A set of sensors required for an ISHS was determined, and interviews were designed based on four factors: perceived comfort, perceived usability, perceived privacy, and perceived benefit. Subsequently, technological trials of the sensor-set followed by two focus group interviews were conducted on nine independently living elderly participants at a senior welfare center in South Korea. Consistent with previous studies, the results of this investigation indicate that elderly participants elicited negative responses regarding usability complexity, and discomfort to daily activities. Despite such negative responses, after acquiring enough awareness about the ISHS’s benefits, the elderly acknowledged its necessity and showed a high level of willingness. Furthermore, these results indicate that for a better adoption of an ISHS, sufficient awareness regarding its benefits and development of elderly-friendly smart home sensors that minimize negative responses are required.

## 1. Introduction

In recent decades, human life expectancy has increased rapidly owing to advances in medical technology and improvement in personal hygiene and nutrition [1]. Furthermore, with the decline in birth rates, the older age group is emerging to gradually represent a greater portion of the population in many countries. Consequently supporting the rapidly growing elderly population is an emerging social challenge [2]. For example, accommodating an elderly population with care institutions puts a financial burden on the government and family members [3]. In addition, it makes the elderly feel isolated from the community and cannot receive emotional support from their family and friends, consequently, having detrimental effects on their emotional state [4]. For this reason, ‘aging-in-place’ has become a trend in which the elderly remain living at home [5]. This enables the elderly to continually play a role as a society member and maintain relationships with their family and friends, thereby having positive effects on the elderly’s emotional status [6].

Despite such benefits, living in a traditional home poses certain challenges for the elderly who adopt aging-in-place. For example, in hazardous situations such as falls, immediate detection and action are impossible in the home environment, thereby threatening the elderly [7]. Furthermore, limited accessibility to professional medical institutions makes it difficult for the elderly to obtain proper healthcare services [8]. Moreover, the elderly are not adept at managing their home energy consumption; they frequently forget turning off lighting and home appliances, thereby wasting energy. Lastly, adverse indoor air quality has negative effects on residents’ well-being [9] and could be especially dangerous for the elderly who are physically weak compared to younger adults. The aforementioned challenges deteriorate the quality of life (QoL) of the elderly, who are adopting aging-in-place; thus, proper solutions need to be established. 

To address this problem, smart home systems are receiving attention, which are residential environments equipped with Internet of Things (IoT) sensors to provide ambient assistance to the elderly [1,10]. For the development of smart home systems, in previous studies, various IoT sensors incorporating both wearable and non-wearable smart home sensors have been applied for the benefits of the elderly-care [11], which can be largely classified into health-related and environmental benefits. For instance, sensors such as cameras [12,13], floor sensors [14], and accelerometers [15,16] have been frequently used in previous studies to detect falls in elderly individuals’ smart homes. Bio-medical sensors such as electrocardiogram (ECG) [17,18], body temperature [19,20], and galvanic skin response (GSR) [21,22] are also applied in smart homes to provide remote healthcare monitoring to the elderly. Power meters and environmental sensors have been used to assist in managing energy [23,24,25] and indoor air quality [26,27] in a smart home. In several studies [1,11,28], an integrated smart home system (ISHS) was proposed because it is essential to improve the elderly’s QoL. Compared to standalone smart home systems, which only provide specific benefits for its residents, the ISHS provides more comprehensive benefits, including health-related and environmental benefits, through the application of various smart home sensors. 

Studies investigating the elderly’s perception of smart home systems have also been conducted because the willingness of the elderly is essentially required to adopt smart home systems [28]. In several studies, the elderly have shown willingness to adopt smart home systems because of their health-related benefits [29,30] such as immediate action in an emergency situation [31], real-time monitoring of physical activity [32], and remote healthcare services [33], all of which assist in maintaining a healthy independent life. In other studies, the environmental benefits of reducing energy costs [34,35,36] through real-time energy consumption monitoring also made the elderly willing to adopt. In contrast, other studies reported that the elderly consider adopting smart homes because of negative responses caused by sensor application, including intrusion of daily life [32,37], physical discomfort [38,39], privacy concerns [30,33], and usability complexity [40].

Although these studies investigated the elderly’s perception of smart home systems, they only reflected the standalone smart home system in their investigation, which only provided either health-related or environmental benefits, based on the limited sensor application. These had limitations in identifying the elderly’s perception of ISHS, which is needed to improve the QoL of the elderly. ISHS provides additional benefits because it comprehensively offers health-related and environmental benefits compared to standalone smart home systems. To achieve additional benefits that are essential for improving the QoL of the elderly, ISHS needs to apply multiple smart home sensors contrary to standalone smart home systems. For this reason, the elderly’s negative responses caused by multiple sensor applications could be different from those of standalone smart home systems. For example, the application of multiple smart home sensors increases complexity usability [41], which leads to fewer adoption of smart home systems [40,42]. In this aspect, the elderly’s perception of ISHS is essentially investigated by reflecting on the changed benefits and possible negative responses. 

Thus, this study aims to conduct the first empirical study to investigate the elderly’s perceptions of ISHS. Consequently, a comprehensive literature review was conducted to determine a set of smart home sensors that are required for an ISHS and to design focus group interviews. The focus group interview was designed comprehensively, evaluating the possible benefits and negative responses of ISHS, which pertains to questions in four categories: perceived comfort, perceived usability, privacy, and perceived benefit. Subsequently, technological trials of the sensors were conducted in the residential homes of elderly participants and focus group interviews are conducted after the technological trials to identify the elderly’s perception of ISHS through qualitative analysis. Based on the investigation results, we made several suggestions to contribute to designing a better ISHS for the elderly.

## 2. Literature Review

### 2.1. Necessity of Smart Home System for the Elderly

Human life expectancy is gradually increasing due to the development of medical science and technology [1], the increasing capabilities in public healthcare systems, and the investment in personal hygiene and a well-being lifestyle [2]. According to this, the entry into an aging society accompanied by a low birthrate in many countries [1,2] has caused many social problems. For instance, assigning a fair budget to provide institutions and hire caretakers to support elderly populations puts a heavy burden on the government. Indeed, the proportion of people aged 80 years or older among the Organization for Economic Co-operation and Development (OECD) countries is expected to increase to 10% by 2050, and spending on long-term care for the elderly is expected to be 3% of the GDP in 2050 [43]. Households also suffer from financial burdens due to excessive healthcare costs for the elderly [3]. 

Moreover, older adults living in care institutions have experienced emotional problems caused by a higher level of dependency and loneliness compared to those living in their homes [4]. It was likewise discovered that “the diminished capacity of self-care additionally influences the decreased satisfaction of the elder’s life” [44]. Above all, it was reported that the elderly prefer to live alongside their families in their own homes [45]. For this reason, “aging-in-place” is preferred by most European nations [5]. The aging-in-place implies allowing the elderly to live in their own homes and depend on others’ help when required [46]. Aging-in-place provides financial benefits, since the elderly remain living in their own homes without using care institutions. Moreover, it reduces social isolation and loneliness among the elderly, advancing independence, and offering emotional support from family members, thereby having positive effects on the elderly’s emotional status [47]. 

Although there are many benefits such as cost-effectiveness and emotional stability in adopting aging-in-place, it should be considered that living in traditional homes is challenging for the elderly. For instance, it can be a significant problem that there is no professional who is responsible for monitoring the elderly’s health conditions and taking immediate actions when accidents occur [48]. Elderly people who live in areas with limited accessibility to hospitals have difficulty receiving proper healthcare services in time [8]. From an environmental perspective, controlling indoor air quality by detecting the level of air contamination is difficult for the elderly. Exposure to indoor air pollution is one of the most serious health hazards that deprive the well-being of many elderly people worldwide every year [9]. In addition, the elderly have difficulty managing home energy consumption because they are not adept at manually controlling home appliances and heating, ventilation, and air conditioning (HVAC) systems [49]. 

In order to support the elderly adopting aging-in-place, smart home systems have been developed. A smart home system is defined as “a residence equipped with various technologies such as network systems, detecting sensors, and appliances that have available automatic controls to provide inhabitants comfort, convenience, and security” [49,50,51,52]. Based on the application of various sensors, smart home systems can achieve environmental [9,28] and health-related [53,54] benefits for the elderly adopting aging-in-place. With the advances in 5G (fifth generation) network systems and sensor technologies, smart home systems have become more efficient and cost-effective solutions to improve the QoL of the elderly [55,56].

### 2.2. Sensor Application in Smart Home System for the Elderly

In previous studies, various smart home sensors have been applied in smart home systems for the elderly’s health-related benefits (fall detection, healthcare monitoring, and activity of daily living recognition) and environmental benefits (energy consumption and indoor air quality management) [10,11]. Falling is an accident that frequently occurs among elderly people, requiring immediate detection to reduce its adverse consequences on the health of the elderly [7]. Cameras [12,57] and floor sensors [14,58] are frequently used for fall detection. Camera captured images were analyzed using a computer vision-based technique to detect falls in real-time [13,59]. Floor sensors detect falls by measuring data that vary in response to movement on the floor [60]. With the advancement of micro electromechanical systems, wearable accelerometers have also been frequently used in fall detection [7,15,16,61]. Acceleration data vary depending on the motion and can therefore be used for fall detection [62]. Shahzad et al. [63] developed a system that detects falls in real time by using smartphone-embedded accelerometers and analyzing acceleration data through machine learning. Similarly, Santos et al. [64] developed a method to detect falls by analyzing acceleration data using a convolutional neural network (CNN), which is a deep learning model.

Sensor-based healthcare monitoring is one of the pivotal health-related benefits of a smart home system for the elderly, which provides remote healthcare services for the elderly by monitoring their health status in real time using sensors [1]. It enables the elderly to take healthcare services in their own homes without visiting healthcare institutions, thereby reducing healthcare costs [8]. For this, diverse sensors such as ECG [17,18,65], body temperature [19,20], and GSR [21,22,66] sensors have been used to continuously measure residents’ bio-signals. Sugathan et al. [67] developed shirts equipped with GSR and body temperature sensors using the Arduino platform for health status monitoring. Lee et al. [68] developed wearable ECG sensors to be attached to a user’s chest for unobtrusive daily health monitoring. 

Recognizing the activity of daily living (ADL) is essential for caretakers to effectively manage their daily lives and detect health problems [48]. Thus, in previous studies, sensor-recognizing activities [69,70] and estimating indoor locations [71,72,73,74] were employed. Ni et al. [75] used depth cameras and analyzed video data using a vision-based activity recognition technique for the recognition of daily activities in the elderly. Awais et al. [76] used accelerometers to recognize the ADL of the elderly, such as sitting, walking, and standing. To estimate the indoor location of the elderly, Morita et al. [77] employed Bluetooth low energy (BLE) beacons in a nursing home. Kim et al. [78] developed a framework to detect depression in the elderly by analyzing activity patterns based on indoor location, estimated using PIR sensors.

In terms of environmental benefits, smart home energy system (SHES) using sensors have been developed in previous studies [79]. The aim of the SHES is to efficiently manage energy consumption while maintaining residents’ comfort based on their energy demands. To this end, power meters and environmental sensors are used, and the sensor-estimated data are analyzed through machine learning for the prediction of energy demands, real-time monitoring, and automation of appliance control [23,24,80]. For example, Filho developed a SHES that monitors the energy consumption of each appliance and provides real-time feedback to residents by using power meters and machine learning [25].

Indoor air quality (iAQ) has significant effects on the well-being of the elderly; therefore, iAQ management systems in smart home environments have been developed. The iAQ system employs environmental sensors measuring parameters such as air temperature, carbon monoxide, and carbon dioxide [26], and the measured data were analyzed for automatic control of HVAC systems and real-time iAQ monitoring for ambient assisted living (AAL) [27]. For instance, Marques et al. [81] developed an iAQ system using low-cost environmental sensors to monitor iAQ in real time using smartphone-based applications for AAL. Similarly, Preethichandra et al. [82] employed environmental sensors to develop an iAQ monitoring system for the automated control of HVAC systems and the detection of hazardous conditions.

In previous studies, various standalone smart home systems have been developed, mostly dealing with specific benefits for the elderly. The benefits include fall detection [7], healthcare monitoring [1,8], ADL recognition [48], iAQ monitoring, and energy consumption monitoring [10,11]. All these factors are essential for effectively improving the elderly’s overall QoL. Because these benefits are closely correlated to the well-being and safety of the elderly in an independent living environment, an ISHS that could provide an integration of these benefits is required. Furthermore, only a few studies have proposed the use of ISHS. Jaouhari et al. [11] proposed an ISHS providing sensor-based healthcare and energy management services to improve residents’ overall QoL. Majumder et al. [1] also suggested that an ISHS provides diverse benefits by integrating multiple smart home sensors in order to make the lives of the elderly independent and active while guaranteeing their safety and comfort.

### 2.3. Elderly Perception on Smart Home Systems

It is crucial to understand the elderly’s perception of using smart home systems [83,84] for its potential benefits to effectively support independent aging and improve their QoL [85,86,87,88,89]. Previous studies have examined how users perceive the benefits of standalone smart home systems [31,40,90,91,92] and the use of smart home sensors [29,32,37,38,93,94,95] to identify the critical factors that influence the elderly’s willingness to adopt smart home systems [33,39,96] through empirical study designs. These studies identified the following as popular factors that influence the willingness of elderly participants to adopt smart home systems: 1. Perceived benefit, 2. Perceived comfort, 3. Perceived usability, and 4. Perceived privacy. The findings primarily describe that the willingness of the elderly to adopt smart home systems was influenced by the perceived potential health-related and environmental benefits. 

In the case of health-related benefits, studies [31,32,33,93] reported that most participants perceived receiving effective assistance for safe and independent living to achieve their goal of optimal aging. These include remote real-time monitoring and sharing of their physical activity levels to potentially helpful entities [32,33] to receive efficient emergency assistance [31] and informing family members to ease their worries [93]. These converge to provide assistance in improving overall health, as the positively perceived benefits of the elderly participants. The perceived environmental benefits that influenced the willingness of the elderly participants to adopt smart home systems were primarily the financial benefit of reducing expenses on household energy consumption, as indicated in previous studies [35,36]. Additionally, by developing a habit of regularly monitoring the energy consumption of household appliances, the elderly participants positively perceived a significant increase in their self-awareness and sense of control [49,92]. This empowered them to pursue a cost-efficient living environment [91], such as turning off unnecessary appliances to reduce electricity expenses.

Conversely, through the application of smart home sensors, many previous studies [36,38,39,49,91,92] have addressed the negative responses derived from the perceived comfort factor to influence the elderly’s unwillingness to adopt smart home systems. Several elderly participants found wearable sensors to be uncomfortable due to the inconvenience of interruptions to their daily routines, such as requiring regular removal of the wearable sensors to recharge the battery and then putting it back on [39], or from other physical discomforts such as itching and irritation [38]. The authors of studies [36,49,91,92] further addressed the negative emotional responses of several elderly participants, such as experiencing discomfort from the inconveniences generated by changing personal behavior habits [36,49,92] which they described as sacrificing their comfort of using convenient household appliances [92] to reduce energy expenses, which was perceived as a significant reduction in their QoL [49]. Furthermore, some elderly participants expressed feelings of guilt and depression [36] due to their lack of control when using appliances that exceed their regular energy consumption, such as heating appliances to stay warm.

In addition, the elderly’s perceived usability has also played a key role in their unwillingness to adopt smart home systems. Pal et al. [40] pointed out that the elderly perceive that the increased usability complexity of smart home sensors leads to the refusal of adoption. In accordance with this, studies [37,38] reported that there was a general consensus on the inadequacy in awareness in the elderly community regarding existing smart home sensors that are associated with the challenges of usability experienced by elderly participants [31,34,35]. Thus, most elderly participants emphasized the need for smart home sensors that require simple user interaction [36,95,96]. Moreover, studies [97,98,99,100] indicate that another significant factor that influences elderly participants’ perception of usability is the instruction or education that is given to them regarding the usage of smart home sensors. For instance, Hu et al. [97] and Tsuchiya et al. [98] reported that the elderly participants perceived a low usability complexity for applications with easy-to-understand instructions and specific guidelines directed specifically toward them. Accordingly, Ashraf et al. [99] pointed out that the elderly experience challenges in usability because the older generation has a decreased prescient and poor literacy; therefore, perceiving the use of smart technology difficult. Consequently, in line with previous studies, Dou et al. [100] also identified that perceived usability complexity would decrease if the elderly are provided with a well-designed guideline and instruction specifically directed toward them.

Another pressing factor that influences the adoption of smart home systems is perceived privacy. Many elderly participants of studies [29,30,32,33,37,49,94] gave negative emotional responses associated with privacy concerns. Misusing data [30,33] by accessing ADL and location information to potentially break into homes and the intrusion into personal life [32,37] violates the elderly’s independence by accumulating continuous real-time monitoring information [29,37], resulting in the question of whether the lack of perceived privacy is worth the financial benefit [49]. Additionally, the authors of studies [29,37,94] have pointed out that several elderly participants perceived using smart home systems meant acknowledging their frailty of old age [94] to themselves and to others [29], which is seen as a stigma rather than the benefits of supporting their health and independence [37]. Nonetheless, these studies focused on investigating the elderly’s perception of standalone smart home systems providing partial benefits [28] rather than fully converged smart homes, defined as ISHS [101,102], which significantly improves users’ QoL [11] by comprehensively providing a more effective and efficient living environment for the elderly. With ongoing advancements in next-generation smart home systems, the deployment of ISHS into homes is imminent.

Through the implications of the factors determined by the application of various smart home sensors, a wider range of benefits, higher usability complexity, and extended privacy invasion will be observed by the elderly, which will undoubtedly generate different positive and negative responses, which play a key role in influencing the willingness to adopt ISHS compared to those from standalone smart home systems. However, despite being over a decade since smart home studies have been a focus of research [41,103,104,105], we are unaware of empirical studies that investigate how the elderly perceive using ISHS comprised of the aforementioned integrated benefits of standalone smart home systems. Therefore, there is a need to investigate the elderly’s perception of ISHS.

## 3. Method

To achieve the objective of this study, we utilized an empirical study design on elderly perceptions of living in an ISHS. For this purpose, an ISHS comprising of the convergence of health-related and environmental sensors was designed, with the purpose of applying a technological trial, followed by focus group interviews, which is discussed in this chapter.

### 3.1. Study Design

#### 3.1.1. Sensor-Set Selection

To design the ISHS, various smart home sensors available in the market were investigated and selected. In this study’s sensor-set selection criteria, we have taken into consideration of four factors: (1) providing comprehensive benefits, (2) low usability complexity, (3) cost-efficient sensors, and (4) minimizing privacy invasion, explained hereafter. Consequently, based on these selection criteria, the smart home sensors used in this study’s ISHS were BLE smart bands, BLE receivers, and two types of environmental sensors.

(1) In this study, we defined a smart home system that provides comprehensive benefits essential for improving the QoL of the elderly as an ISHS. Based on a literature review, essential benefits that are commonly considered as important benefits of smart home systems for the elderly are as follows: fall detection [7], healthcare monitoring [1,8], ADL recognition [48], iAQ monitoring, and energy consumption monitoring [10,11]. Thus, the sensor-set for the ISHS were to provide the essential benefits of a smart home system.
(2) The sensor-set selection employed the integration of as few smart home sensors as possible since the consideration of usability complexity is a critical factor that determines the willingness of elderly users [40,42].
(3) By minimizing the applied sensors for our ISHS, the cost factor of our sensor-set application can be efficiently minimized; we assured the sensor-set selection provided the collection of the necessary data that can be analyzed to provide the aforementioned essential benefits for the elderly in an ISHS environment, despite minimizing the number of applied smart home sensors.
(4) To minimize the privacy invasion of the ISHS user, the sensor-set selection excludes the application of sensors which can record real-time visualizations. For instance, indoor location information can be easily acquired through sensors integrated with cameras, however, due to the nature of privacy invasion using cameras, BLE beacons were chosen as our sensor for acquiring indoor location information.

**Figure 1:**
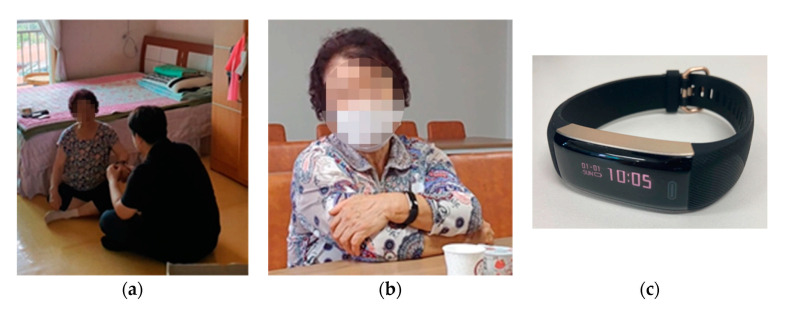
Application Wearable BLE beacon (PWB-400) (**a**) Tutorial prior to technological trial, (**b**) Wearing of BLE beacon, (**c**) Wearable sensor model (PWB-400).

The BLE smart band (Figure 1c PWB-400) was selected to provide the benefits of fall detection and healthcare monitoring. The BLE smart band is a wrist-worn sensor integrated with a 6-axis accelerometer to track the movement of the sensor and a heart-rate monitor so that its users can view their physical activity information (1. Heart-rate, 2. Step count, 3. Walking distance, 4. Calorie consumption, and 5. Sleeping patterns) in real time with a low usability requirement [39,93]. These two functions enable the BLE smart band to detect falls by tracking the sensor movement data via accelerometers [15,16] and monitoring the healthcare of the elderly user. Originally, the BLE smart band was designed for personal use only; the only entity with access to viewing the sensor data was the user. Furthermore, the users were required to connect the device to a single compatible smartphone within a short range to access and view the collected sensor data. However, given these two limitations, a real-time IoT-based sensor monitoring corporation provided a convenient solution by modifying the PWB-400s’ firmware to act as a wearable BLE beacon, allowing the sensor data to be stored directly onto a cloud server via the corporation’s compatible BLE receivers.

**Figure 2:**
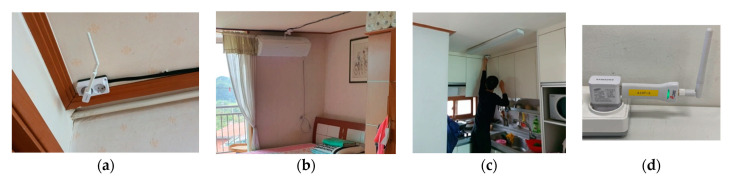
Application of BLE receivers (**a**) Installed BLE receiver, (**b**) Installation above participants’ bed, (**c**) Installation in the kitchen, (**d**) BLE receiver model.

These BLE receivers (Figure 2d BLE Receiver) acquire sensor data, including the indoor location of the wearable BLE beacon when in close proximity, which is then processed and displayed onto their developed web-based platform. Through this platform, both the elderly user and the research team can monitor the health-related information and indoor location in real time with access to the internet, without the need for the specific compatible smartphone. However, there may still be privacy issues regarding the collected sensor data, therefore, we have provided the elderly participants with a privacy policy which ensures their collected data will not be accessed by other entities such as, caretakers, family members, and medical professionals, unless the participants themselves have consent for approval to share with them. Thus, with the approval of elderly users, the sensor data can also be accessed by other entities through a web-based platform on any device. 

Subsequently, through this sensor-set’s wearable BLE beacons [77], BLE receivers and its integrated accelerometers [76], the elderly user’s ADL recognition can be acquired by analysis of the sensor’s collected user’s indoor location information [71,72,73,74] and activity recognition [69,70] (which are significantly closely associated to each other). The daily activities which can be recognized through sensor data include sitting, walking, sleeping, watching TV, cooking, bathroom usage, and etc. [77]. The purpose of the recognition of these daily activity provides an automatically generated report of the elderly’s daily profile, which can potentially be used to effectively improve their QoL. For instance, through the elderly’s daily report, the caretakers can effectively manage the elderly’s lives to improve their QoL without requiring manual write up of the daily report through constant supervision. Additionally, the reports can be evaluated by medical professionals to remotely diagnose for potential mental health-related disorders such as depression [78]. Furthermore, through the application of this sensor-set’s wearable BLE beacons and BLE receivers, the ADL recognition features for the necessary data collection is provided at a significantly lower price compared to other wearable sensors such as Fitbit, Apple watch, Samsung Gear, and others available in the market. Additionally, because of the benefits provided by these two sensors, other commonly used smart home sensors, such as bed sensors and contact sensors, were not considered for the final selection.

**Figure 3:**
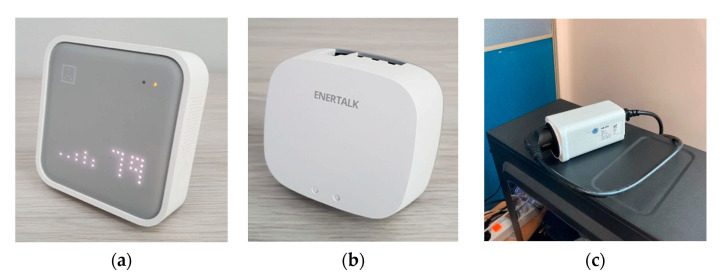
Environmental sensors (**a**) Awair Omni sensor, (**b**) Fuse box smart meter (Enertalk ENCORDED), (**c**) Individual household appliance smart meter (Enertalk Smart Plug).

One of the environmental smart home sensors is the iAQ sensor (Figure 3a Awair Omni), which provides the benefit of iAQ monitoring. The iAQ sensor measures the iAQ by processing five different environmental indicators (1. Temperature, 2. Humidity, 3. CO_2_, 4. Volatile organic compounds (VOCs) and 5. Dust) and outputs the overall iAQ score (range = 0–100) on the sensor display, which is easily viewable by the elderly. This smart home sensor has a single button on the side, which when pressed once, displays each environmental indicator one at a time, providing a low level of usability for the elderly. The sensor data are collected in real-time at every 1 min interval, which is stored on to a cloud server also accessible by entities other than the elderly user.

The smart meters (Figure 3b Enertalk ENCORDED, and c Enertalk Smart Plug) provide the benefits of the users’ household energy consumption monitoring. These smart meters measure the energy consumption of both individual household appliances and total household consumption in real time and store the data on a cloud server, which can also be accessed by other entities. The energy consumption of appliances is monitored by plugging in the power chord of each appliance to a smart meter, which is then plugged into the power socket. Furthermore, smart meters can also detect the household appliances’ anomalous behaviors [106], thereby helping determine which appliances may be faulty and cause consumption of excess energy. In addition, smart meters are unobtrusive smart home sensors, which, as mentioned in Section 2.3, provide potential benefits without generating possible negative emotional responses from self-monitoring energy expenses when using household appliances. 

Every sensor-set network is specially designated to each elderly participants, that is, each sensor-set is paired to its designated elderly user’s home wireless internet router. The collected sensor-sets’ data is transmitted to a single server and processed to display the necessary information, which can be used by both the elderly users and other entities. As a result, this selection of our cost-efficient and minimized sensor-set covers a wider spectrum of smart home benefits, which defines a definite difference from the standalone smart home system’s sensor-sets identified in previous studies. Therefore, this sensor set was selected over other smart home sensors for ISHS design in this study.

#### 3.1.2. Technological Trial

The study setting was within a residential apartment for the elderly population, which is located within the senior welfare town in Gim-Je province, South Korea. For an accurate investigation of the elderly’s perception, we conducted a technological trial of the sensor set. The sensor type included in the sensor set is classified into wearable sensors (wearable BLE smart band) and non-wearable sensors (BLE receivers, iAQ sensors, and smart meters). In the case of wearable sensors, since the elderly have to physically wear them, it is essential that they experience it first-hand while performing their regular daily activities through a technological trial. Therefore, each elderly participant was given a wearable BLE beacon and were instructed to wear them for a duration of 24 h to provide them with the experience of applying the sensor to their independent living. In addition, because the wearable BLE beacon requires the BLE receivers to operate, the BLE receivers were installed on the ceiling of each participant’s actual residential homes. 

The households were comprised of three different dimensions: (1) A-type (N = 3, 3500 mm × 8500 mm), (2) B-type (N = 4, 4300 mm × 9500 mm), and (3) C-type (N = 2, 6400 mm × 9350 mm). In the A-type and B-type households, seven BLE receivers were installed on the ceiling, as shown in Figure 2a, of which four were in the bedroom, one in the kitchen, one in the bathroom, and one at the entrance to the household. However, C-type households were larger; therefore, 10 BLE receivers were installed: three in the first bedroom, one in the second bedroom, two in the living room, one in the dining area, one in the kitchen, and one in the bathroom. During the installation process of the BLE receivers, one member physically installed the sensors (as shown in Figure 2c), another member set up the connection of the sensor set to the cloud servers, and the last member provided a brief introductory session that explained the sensor set (what the sensors measured, how the data was measured and accessed, how the sensor battery was recharged, and the possible benefits that could be acquired from the sensor data) to the elderly participants to help them better understand this research investigation, as shown in Figure 1a. 

As the sensors were applied to the actual households of the participants, the participants were not given instructions to perform any specific activities that intentionally deviated from their regular activity patterns during the duration of the technological trial because this study aims to understand how the elderly perceive the sensors in an actual living environment. Therefore, the participants only performed activities within the boundaries of their normal routines, such as cooking, watching television, socializing with their neighbors, outdoor exercise, showers, and sleeping.

In the case of the non-wearable sensors (iAQ sensor, smart meters), the elderly participants were subjected to a technological trial to test the environmental sensors during the two focus group interview procedures (1 h 30 min and 1 h 15 min, respectively). The participants were all given the opportunity to sufficiently examine and use each sensor, as shown in Figure 3. During the trial experience, the investigators provided a detailed explanation of the non-wearable sensors’ functions and features, such as how they are operated, how they measure the environmental data, what type of environmental data is collected, and what possible benefits the participants can experience from the collection of the environmental data. 

### 3.2. Focus Group Interview Design

#### 3.2.1. Interview Design

**Figure 4:**
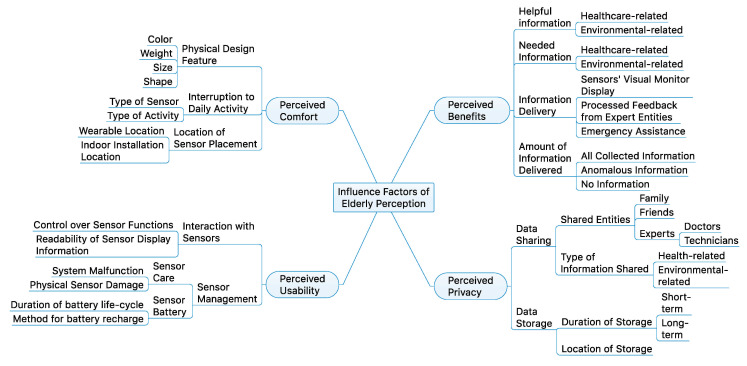
Interview categories for this study’s elderly perception investigation.

To develop a guideline for the group interview questions, categories pertaining to the four major categories determining the influencing factors of elderly’s perception of ISHS were organized (as shown in Figure 4). These categories were the most common factors of influence, as reported by the authors of the studies identified in Section 2.3: 1. Perceived comfort, 2. Perceived usability, 3. Perceived benefits, and 4. Privacy. It is also important to note that, of these factors, perceived comfort, perceived usability, and perceived benefit derived from key constructs that positively relate to direct determinants of users’ perceived satisfaction and intention to use in theories of acceptance of technology, such as the Technology Acceptance Model (TAM) developed by Davis et al. [107] and Unified Theory of Acceptance and Use of Technology (UTAUT2) developed by Venkatesh et al. [108], which is derived from the original TAM. These technology acceptance models were used as a basis to evaluate the factors of influence in many previous studies on elderly perception of standalone smart home systems. Therefore, this study’s interview design employed the identified factors from previous evaluations to analyze the elderly’s perception of ISHS.

The interview design was a semi-structured interview with predefined guideline questions, as shown in Appendix A. The purpose of the guideline questions referring to the aforementioned factors of influence was designed to assist group discussions in providing topics for elderly participants. Furthermore, the structure of the questions helps to provide the elderly participants with as much scope as possible for their responses, while helping to lessen the pressure in adjusting to the gradual difficulty of the in-depth exploration of their perception. The guideline questions’ assumption purposes are clarified in the following.

Guideline question 1 aims to discuss the first category of how the elderly participants perceive the physical and psychological comfort of the wearable and non-wearable smart home sensors. This factor is associated with the constructs of TAM’s perceived ease of use (PEOU) and UTAUT2’s hedonic motivation, which are defined as the user’s perceived satisfaction [109] and pleasure derived from the experience of using technology [110]. Therefore, the relevant discussion topics associated with the predefined questions elicit both how the participants felt through this experience and their preference for future implementations regarding the ISHS smart home sensors’ physical design features (size, weight, and color), interference and inconvenience to daily activities (indoor and outdoor), and sensor wearables and installation locations. 

Guideline question 2 aims to discuss the second category of how elderly participants perceive the usability of the ISHS sensor set. This factor associates directly to TAM’s PEOU and UTAUT2’s effort expectancy (EE) which are defined as the user’s perceived satisfaction and complexity level of the applied technologies [108]. This factor considers the control that the participants have over using the ISHS sensor-set functions (interaction), the difficulty level of understanding the displayed sensor information, their perceived worries and concerns about sensor damage, inconvenience regarding battery life, and preference of these aforementioned topics in future ISHS implementations. 

Guideline question 3 aims to discuss the third category of how elderly participants perceive the invasion of their privacy within an ISHS environment due to the collection and storage of their personal information through ISHS monitoring and how they perceive sharing this type of personal information acquired, and to specific entities (family, friends, and experts). Although this factor is not directly associated with constructs of the technology acceptance models, many previously identified studies on elderly perception of standalone smart home systems have reported that the value of importance privacy plays a role in influencing the willingness to adopt.

Guideline question 4 associates with the perceived benefits category, which aims to discuss how the elderly perceive the benefits of the various types of information that can be acquired from a wider range of ISHS monitoring with regard to which type of information (specific information types pertaining to health-related and environmental monitoring) they perceive the most helpful and useful. This factor directly associates to TAM’s perceived usefulness (PU) which is defined as “users’ assessment of whether employing a specific product or service can improve their job performance and efficiency” by Davis et al. [107]. This factor further aids in examining the relationship between users’ perceived satisfaction and intention to use, which can also be described as a key determinant factor in understanding the elderly participants’ willingness. In addition, this includes examining the method in which participants prefer to receive information (visual aid, verbal communication, and physical action), and how much of the information they want to know. Moreover, this category concludes by exploring the consideration of all the aforementioned discussion topics to elicit a final response that determines the intention and willingness to adopt an ISHS. Thereafter, in line with this concluding response, participants were asked for any further improvements or preferences for future implementations for ISHS, including limitations that were not addressed in the group discussions.

#### 3.2.2. Participants

Nine independently living participants who volunteered for this study were recruited and rewarded with gift coupons and red ginseng drinks for their participation. Of the nine participants, only eight participants proceeded with the first technological trial; one participant had changed her mind and was thus unwilling to participate in the first technological trial. However, the participant wished to contribute to both the second technological trial and focus group interview, and so participated in the second interview session. As a result, data from all nine participants (nine females) were included in the analysis. Participants’ ages ranged from 68 to 87 years (average = 78 years) and were all Asians. All participants provided informed consent for inclusion before participating in the study. The study protocol was approved by the 157th deliberation of the Korea University Bioethics Committee (approval code: KUIRB-2020-0086-01). 

#### 3.2.3. Focus Group Interview & Procedure

For qualitative study designs, conducting focus group interviews generates a rich dataset for exploratory research and development [111]. Focus group interviews are usually conducted with a group size of minimum four people, up to fifteen people, to provide a better moderation of group discussions [33,34,112]. Thus, we determined the number of participants in this study to be nine. Although the sample size of this study may seem insufficient to represent the elderly population, according to Nielsen et al. [113], five participants were adequate to address at least one issue regarding the overall usability that could be discovered with a larger sample size. 

**Figure 5:**
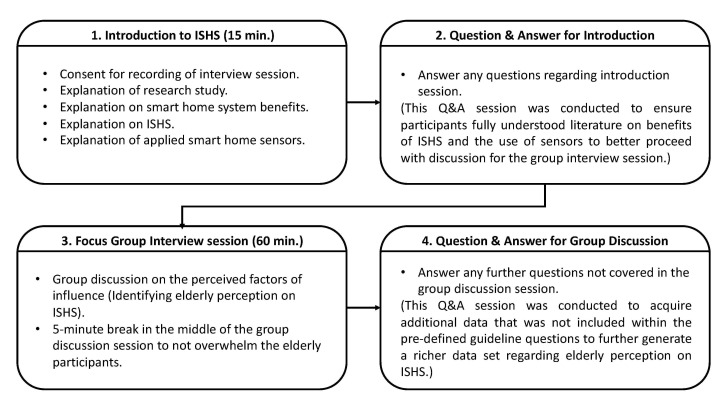
Focus Group Interview procedure.

The focus group interview procedure comprises four sections, as shown in Figure 5. To ensure that the same level of group discussions was conducted through both interviews, the same information of the focus group interview procedure was provided to both groups. Each focus group interview session began with an introductory session incorporating a presentation with visual aid to provide the elderly participants with comprehensive knowledge on the privacy issues and a wider range of benefits provided by an ISHS in comparison to those of current standalone smart home systems. For instance, the elderly participants were given sufficient explanation about what type of health-related and environmental monitoring data are collected, where the data are stored, who has access to it and how they can access it, and how these data will be used to provide the benefits of an ISHS to improve their QoL. In addition, the participants also received further explanation about the smart home sensors used in this study’s sensor-set to ensure they acquire adequate knowledge on the usability, such as how the sensor-set functions work and how to charge their batteries. After the introduction session, both groups were given the opportunity to ask any questions regarding the explanation that required clarification until they were ready to proceed with the group discussion session, which focused on the elderly participants’ perceptions of the ISHS. 

It is important to mention that this introductory session was specifically included in the design to ensure the elderly participants acquired sufficient knowledge on ISHS through both technological trials to understand that the purpose of the predefined guideline questions were directly associated to perception on ISHS and not for the standalone smart home systems.

**Figure 6:**
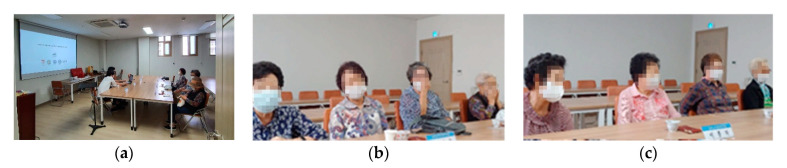
Focus Group Interview sessions (**a**) Conference room, (**b**) First group, (**c**) Second group.

The focus group interviews for this study were conducted on 18 September 2020, in a conference room provided by facility members, as shown in Figure 6a. Participants were divided into two focus group interview sessions, as shown in Figure 6b,c, where the first group comprised four participants and the second group comprised five participants. Each interview group was structured with participants who were familiar with each other so that during the discussions, they would be more open to freely express their ideas and feelings [111]. The first group interview was conducted at 11:00 in the morning and the second at 14:00. The group interview sessions took 1 h 30 min and 1 h 15 min, respectively. A 5 min break along with snacks and drinks during the group interview sessions was provided to both groups so that the participants do not feel too burdened or tired. During the focus group interviews, there were two moderators and two assistants. The moderators led both the introductory sessions and group discussions, while the assistants managed the technical support involving computer-assisted visuals to better aid the elderly participants in understanding the ISHS and its benefits. 

## 4. Results

**Figure 7:**
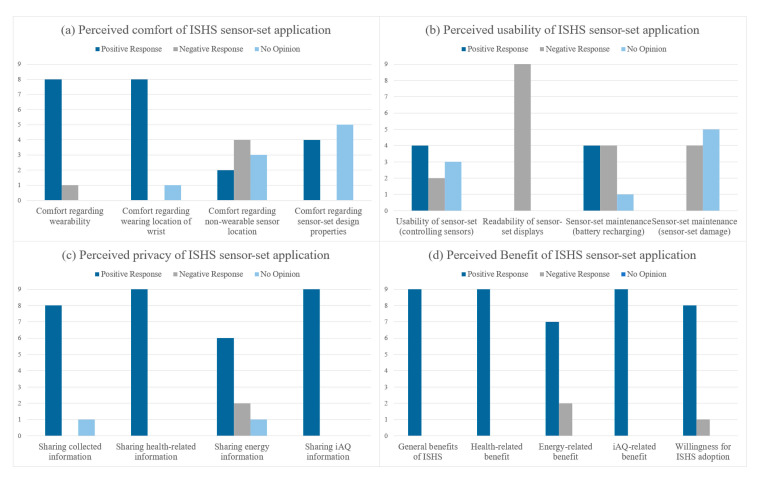
Summarized representation of group discussion results (**a**) Perceived Comfort, (**b**) Perceived Usability, (**c**) Perceived Privacy, (**d**) Perceived Benefit; *X*-axis consists of relevant discussion topics of each factors of influence; *Y*-axis depicts the number of participants who responded.

The present study examined how elderly participants perceived using an ISHS through two technological trials and follow-up focus group interviews. Two focus group interview data (1 h to 1 h 15 min) were analyzed using a qualitative approach. The analysis of the data was performed by the lead investigator, and the validity of the interpretations was examined by other members of the research team. A summarized representation of the results from the group discussion responses by the participants is shown in Figure 7.

The findings regarding the perceived comfort (Figure 7a) of this study indicate that the elderly participants experienced both comfort and discomfort with the ISHS sensor-set. In particular, eight of nine participants perceived the wearable BLE beacon comfortable to wear on their wrists. On the contrary, more participants elicited negative responses regarding the comfort of non-wearable sensor locations. Despite the comfort factor of the wearable BLE beacon, in regards to interruptions to daily activities, five participants mentioned experiencing discomfort from using the wearable BLE beacon. For instance, discomfort was experienced during activities involving getting their hands wet such as dishwashing, washing hands, and taking a shower. The four negative response generated from non-wearable sensors was specific to iAQ sensors, that is, the potential discomfort from the inconvenience of iAQ sensor’s placement on the dining table. Furthermore, there was no physical discomfort experienced from the design properties of the ISHS sensor-set.

The findings regarding the perceived usability (Figure 7b) indicate the elderly participants experienced difficulty with this study’s ISHS sensor-set. Two participants explicitly mentioned negative responses regarding the difficulty in usability of the sensors, and all participants responded negatively regarding the readability of the sensor displays. For instance, their perceived difficulty with the interaction (control of sensor functions and response time) of wearable BLE beacon and iAQ sensor were relatively easy, however, understanding the displayed information was perceived very difficult. The negative responses were generated from the small and difficult-to-read text size of wearable BLE beacons and the use of symbols to describe the iAQ information. For the sensor-set maintenance, many participants mentioned difficulty with recharging the batteries of the sensor, and negative emotions generated from the worries of damaging the sensors during their daily activities. It is worthy to note that all the participants mentioned their preference of using long-lasting replaceable batteries instead of the current method requiring recharging sensor batteries using cables.

On the contrary, the elderly participants generally perceived the privacy factor positively (Figure 7c). Eight participants strongly mentioned that they positively perceive sharing their collected information while one participant provided no response. The participants who were willing to share their information indicated they were willing to share with all entities who could provide assistance to the safety of their independent living such as, friends, family members, caretakers, and medical professionals. In addition, all participants mentioned they were willing to share health-related and iAQ monitoring information, however, two participants explicitly mentioned they were not willing to share their energy consumption monitoring information to anyone else.

Similar to the results of perceived privacy, the elderly participants perceived the benefit of ISHS (Figure 7d) very positively. All participants mentioned the benefits of ISHS is very helpful for people adopting independent aging. Among the ISHS benefits, the participants also mentioned that health-related and iAQ-related benefits are the most necessary benefits an ISHS should provide. In the case of energy-related benefit, two participants perceived it was not a necessary benefit that should be applied in ISHS. Consequently, when asked if the elderly participants were willing to adopt an ISHS, all but one participant mentioned their unwillingness to adopt an ISHS. 

## 5. Discussion

A comparative discussion analysis was applied to identify and document common factors of influence and frequently addressed topics and issues in comparison to standalone smart home systems for each interview’s transcript data and additional notes (taken throughout the interview sessions). The primary findings of this study demonstrate that the elderly perceptions associated with the aforementioned four factors were quite similar to those of previous studies, despite the standalone smart home systems of previous studies employing a narrow selection of smart home sensors to provide a specific benefit in contrast to our comprehensive ISHS, which employs a wider range of various smart home sensors to provide a significantly larger scope of different levels of benefits. Among many similarities, in particular, the perceived benefit of smart home systems outweighed the other factors influencing willingness to adopt since the need for assistance in independent aging was significant for elderly users, and the higher preference of employing wearable BLE beacons for health-related benefits. Despite finding similarities in elderly perception, it is important to note that, because ISHS employs a wider range of smart home sensors for comprehensive benefits, the emphasis on the usability complexity and benefits of the ISHS was significantly greater than that of standalone smart home systems.

This section will be divided into four subsections associated with the factors of influence introduced in the interviews that elicit the elderly participants’ responses on their perceived comfort, perceived usability, perceived privacy, and perceived benefits of ISHS. 

### 5.1. Perceived Comfort

Previous studies on standalone smart home systems indicated that elderly participants perceived the physical comfort of the wearable sensors more positively when worn on the wrist [93,95] as compared to neck-worn, waist-worn, or head-worn. Fang et al. [93] and Qi et al. [95] further indicated that elderly participants preferred the wrist for the wearing location because it is more comfortable than other locations and only requires minimal effort to view the sensor information. As expected, the findings in this present study were congruent with the aforementioned findings, where most participants (*n* = 8) also perceived the physical comfort of wrist-worn wearable BLE beacon positively because of its comfortableness and ease in readability of the sensor display information. In addition, when asked about their preferences for alternative wearing locations for the sensor, the participants responded that because they were so used to wearing bracelets and watches for several decades, the wrist was the most optimal location. “*The older generation these days are so used to wearing things on our wrist since we were young, and so there is no burden with wearing this (referring to wearable BLE beacon) on our wrist now.*” (Participant 3) Furthermore, one participant compared the wearable BLE beacon and her smartphone to point out how the sensor requires significantly less effort when trying to view the current time as it only requires a single touch on the sensor display, “*Using smartphones requires considerable effort since I have to take it out of my bag, open the smartphone safety case, and press the on button to light up the screen, but this device, all I have to do is look at my wrist*.” (Participant 4).

However, despite the positive perceived comfort of the wearable location, five participants mentioned experiencing physical discomfort from the inconveniences and interruptions to daily activities, primarily activities related to getting their hands wet or sleeping. This negative response was generated because the participants were instructed to wear the sensor at all times to ensure continuous data collection. In our study, participants mentioned experiencing concerns regarding whether they needed to take off the sensors when they had to get wet, such as dishwashing or bathing. This finding was in line with Reeder et al. [32], which indicated that several participants mentioned the same issues with regards to getting wet. Additionally, the participants who mentioned being used to wear a watch further mentioned not being able to take off the sensors before sleeping generated inconveniences that interrupted their regular behavior patterns. This finding was also apparent in a study by Farina et al. [39], where most participants reported that they experienced interruptions to their daily activity pattern when they were unable to take off the wearable sensor before sleeping. 

In this regard, elderly participants often suggested taking off the sensors when sleeping because they experienced discomfort from interruption to their normal behavior patterns. However, because elderly individuals are prone to critical health conditions during sleep [114], it is important to continuously monitor physiological levels even during sleep. Therefore, we suggest an alternate smart home sensor that can continuously monitor the physiological levels of the elderly while generating no discomfort from wearable sensors. This can be achieved through the use of non-wearable sleeping sensors that collect the same set of information but are placed under the mattress. 

The participants of our study further reported inconveniences associated with their perceived discomfort of interruptions that could arise in daily activities depending on the placement of the iAQ sensors. For instance, the participants mentioned that they would experience interruption if they were placed anywhere within the hands reach, such as dining tables. “*I would really like it if it (referring to iAQ sensor) was placed somewhere else, somewhere where my hands won’t reach because it’s uncomfortable having to move the sensors if it’s in my way of doing something.*” (Participant 1) For this reason, an ideal iAQ sensor deployment within an ISHS would be on the walls of the kitchen or central hallway at eye level to minimize inconvenience while still being easily visible for monitoring. The suggested preference for sensor placement takes into consideration the findings from Hargreaves et al. [91], which indicate that elderly participants preferred the placement of non-wearable sensors on the wall of the kitchen, hallway, or lounge, such that they are still visible from a distance, but are out of reach. 

In the case of the smart meters in our study, the participants did not explicitly mention any physical or psychological discomfort. This finding is different from previous studies that found psychological discomfort from the negative responses generated due to the availability of real-time observations of their household energy consumption habits [36,91,92]. This is because in our study, we employ non-visual display energy smart meters, in contrast to the use of visual display energy smart meters, which were used in the previous studies. Furthermore, with regard to this, when asked our participants if they preferred to visually observe their household energy consumption, they said that they did not prefer seeing them personally. Thus, the findings suggest that the use of non-visual energy smart meters would be effective in reducing the psychological discomfort related to the household appliances energy consumption monitoring.

### 5.2. Perceived Usability

The elderly population’s perceived low complexity in smart home technology usability is a critical factor of influence that is associated with the adoption rate of smart home systems, as indicated by the findings of previous studies [31,37,40,42]. In addition, studies by Qi et al. [95] and Vincent et al. [96] discussed that the elderly population emphasizes the requirement of a simple user interaction. These aforementioned findings were in accordance with our results, which indicate that the elderly participants significantly value and require low perceived complexity when using smart home sensors. In particular, one of the most challenging experiences reported by all the elderly participants was understanding the different information displayed on the IAQ sensor. Several participants elicited negative responses to low perceived ease of use. Because the younger generation is exposed to and incorporates technology within their everyday lives more frequently than the elderly, more elderly users generated negative emotional responses from their frustration and difficulty in understanding the sensors, which was in line with the findings of a study by Brown et al. [36], who reported that unlike the younger generation participants, most elderly participants generated negative emotional responses due to the difficulty in understanding the display information. 

Additionally, two participants expressed frustration from the difficulty in controlling the response time of the displayed sensor information on the BLE smart bands by reporting that the display turned off too fast before they could press the button to view other information. The aforementioned findings can be explained by the lack of awareness of smart home sensors among the elderly participants in our study. Findings by Coughlin et al. [37], Kekade et al. [38], and Barnicoat et al. [35] reported that there is a general consensus that there is inadequate awareness among elderly participants, resulting in a low rate of actual usage of smart technologies, suggesting a need to raise awareness. As expected, the results obtained in our study were well aligned with this finding; only two of the nine participants were aware of and experienced in using smart technologies; thus, the remaining seven participants did not have prior experience or knowledge and perceived the usability of smart home sensors more difficult. 

One participant explicitly mentioned that because she was not aware of smart home sensors, she perceived the usability of an ISHS very complicated and was very hesitant to continue using an ISHS, “*I find it difficult to use these technology, so I am not very confident I could properly use them in my life*.” (Participant 1) This was similar to the study by Mihailidis et al. [115], suggesting that several participants were not comfortable with the usability of technology and thus were hesitant to use them. In contrast, the rest of the participants in our study generally perceived the usability of various sensors positively. Additionally, for a reasonable number of functions available as part of the sensors, the usability factor of an ISHS was perceived as positive. Our results also indicate that the participants found it very inconvenient to charge the batteries of the wearable BLE beacon and IAQ sensors using a charging cable. In particular, burdening the elderly participants on requiring them to take off the wearable BLE beacon every 1–3 days to charge the batteries, “*We find this type of charging difficult. Once every three days is enough, but at our age, we tend to forget things. Honestly, I would really prefer if we could just replace the batteries once a month instead of having to recharge every few days.*” (Participant 7) This was also consistent with the findings of Farina et al. [39] and Kekade et al. [38]. 

In relation to this issue, one participant pointed out, “When I forget to charge the battery, I feel a sense of anxiety for the inconvenience where I have to choose between not going outside without the device which means I can’t monitor my activity levels or stay home and wait for it to be charged.” (Participant 5) Additionally, most participants mentioned how they forgot to remember wearing the wearable BLE beacon after charging, generating negative emotional responses for the missing monitoring data. This finding is similar to the findings of a study by Reeder et al. [32], in which several participants faced the challenge of remembering to wear the sensors that contributed to the missing data. Consequently, all participants in this study perceived using long-lasting batteries as an important factor contributing to the usability of smart home technology. This finding confirms the findings of other aligning studies by Qi et al. [95] and Jang et al. [116], who reported that elderly participants emphasized the value of smart home sensors with a long battery life. 

It is important to recognize that the elderly participants also perceive the repairs and follow-up services as another important variable contributing to the usability of smart home sensors. The results of our study indicate that elderly participants worry about damaging the sensors or having the sensors malfunction for reasons unknown. These results are in accordance with those of Vincent et al. [96] and Balta-Ozkan et al. [49]. As a result, it has often been reported by the participants that the insurance and availability of repairs in the form of follow-up services is an important variable for consideration when adopting smart home systems. Overall, the interface of the sensors’ displays information and the method of charging the sensor’s battery were considerations that elderly participants frequently suggested for change in terms of their perceived usability. For instance, all participants expressed that the iAQ sensor’s display interface, which uses shapes as indoor environment condition indicators, were complicated to understand and that using charging cables generates stress from worries of having to often recharge. Because the integration of various sensors increases the usability complexity, it is essential that the sensors are applied to an ISHS to generate minimal discomfort, such as using text instead of graphics to display information for ease in understanding sensor displays and using sensors powered by lightweight replaceable batteries.

### 5.3. Perceived Privacy

Several findings from previous studies indicate that many elderly participants perceived the invasion of their personal privacy as a very significant factor influencing the adoption of smart home sensors [32,37,49,90,117,118]. Conversely, there were more findings from other studies that indicated most elderly participants perceived invasion to their privacy as much less significant compared to other factors of influence [29,30,31,34,38,95,119,120] such as usability and benefits. In this study, despite ensuring that the participants were well aware of the privacy issues generated through an ISHS environment, the results obtained in our study align with the findings that report the latter: most elderly participants consider invasion of their privacy significantly less as a factor of influence, “*Older adults like us these days don’t understand how to read these information. For us, it is much better for our neighbors, friends, or family to know about these information so that they can help us out instead. But, since family and experts of these field tend to be far away, we share these information we each other (referring to neighbors) so they can help us out.*” (Participant 4) A similar finding by Kim et al. [30] reported that privacy is less of a concern to the elderly population as compared to the younger generation in South Korea. However, finding was shown to be influenced by education and age; more educated participants were less likely to share their information. Given that the younger generation in South Korea tends to be more educated [121], they were more concerned about privacy invasion than the elderly. Additionally, a quantitative data analysis by Singh et al. [122] indicates that the Asian elderly population perceives privacy concerns as less significant compared to the perceived benefits. 

Two participants explicitly pointed out their concerns about the invasion of their privacy from the installation of BLE receivers at the beginning. Despite informing the participants, the BLE receivers were not capable of video or audio recording features, and were still initially worried that BLE receivers would be able to analyze their physical postures when changing clothes and listening to their conversations, “Even though we were ensured they (referring to BLE receivers) did not video record, I was worried that this was monitoring my body postures while I was changing my clothes this morning.” (Participant 4) This finding was congruent with other research findings by Reeder et al. [32], Chung et al. [117], and Chung et al. [118], who reported that the elderly participants’ concerns of sensors were able to detect their specific action through body postures. Moreover, Chung et al. [118] further indicated that several elderly participants were hesitant to install sensors inside their bathrooms, as they perceived it as a significant intrusion to their personal privacy. Conversely, the findings of a study by Mihailidis et al. [115] pointed out that elderly participants perceived the installation of sensors within the bathrooms acceptable and were generally supportive of doing so. In the case of our study, the findings are parallel to the findings of Mihailidis et al. [115], which can be explained by how elderly participants perceived the bathroom to be a place where accidents were most likely to occur. 

The findings of how elderly participants’ perceived data security and sharing of collected personal information varied among previous studies. Reeder et al. [32] and Demiris et al. [120] found that several elderly participants had privacy concerns regarding the security of their collected personal information; concerns about access to their daily activity patterns, such as misusing information to determine when the participants’ homes were vacant. However, other participants of the same studies [32,120] and other studies by Wild et al. [31] and Parag et al. [34] indicated that participants were willing to share their personal information with potentially helpful entities, primarily to receive more effective monitoring. In the case of our study, the results obtained were consistent with those of the latter. Furthermore, the elderly participants in our studies reported that the cause of their concerns was not being able to share their information with others, primarily their location information; they perceive that sharing their personal information is used to potentially aid them in living a safer and independent life. “What we are afraid of is not from sharing our information, it’s actually from not being able to share our information, because if something happens to us, no one will notice. It’s because someone is monitoring us that gives us assurance of our safety.” (Participant 4) This finding is also associated to the factor on how all the participants acknowledged their frailty in old age; therefore, they willingly shared their personal information, daily activity patterns, and left their doors open at all times so that their neighbors could freely stop by and check their health condition, which was an unexpected finding. 

In comparison to our findings, the findings of Coughlin et al. [37], Demiris et al. [120], and Hall et al. [94] indicated that most elderly participants negatively perceived using smart home sensors because its adoption meant acknowledging their frailty of old age. It should also be noted that all the participants in our study suggested that the storage of their collected personal information over a long period of time be used to effectively monitor and understand the changes in their health conditions, whereas the participants of the study by Coughlin et al. [37] expressed their concerns about the accumulation of their collected personal information. Moreover, all elderly participants in our study elicited positive responses on how they perceive that using obtrusive monitoring sensors provides assurance to their safety in independent daily living, easing their worries about both healthcare and environment management. 

For instance, one participant stated, “Because they (referring to smart home sensors) are there, we can see them, and because we can visually see them, we feel assured that our health and living environment conditions are being monitored. If we could not see them, we would forget that they are even there, and so we would not know if it is doing its job.” (Participant 5) This was an unexpected finding, which was inconsistent with previous studies by Hargreaves et al. [91,92] and Brown et al. [36], which indicated that the obtrusiveness of the sensors generates negative emotional responses from the elderly participants by creating a sense of anxiety, fatalism, and despondency. Although privacy issues were not a concern in this study’s findings, it is important to mention that the invasion of personal privacy was generally perceived negatively in previous studies. Additionally, privacy was perceived differently between elderly populations of different nationalities, indicating that it is less of a concern among Asian nationalities [122]. Thus, we believe that the findings would be different if a wider range of nationalities among elderly participants is to be employed. Regardless of the similarities and inconsistencies between the findings of previous and current studies, it is critical that strict policies and clearly defined guidelines regarding terms of privacy are presented to assure the elderly participants that their data are safe and secure and will be under no circumstances ever be misused.

### 5.4. Perceived Benefits

When asked about the perceived benefits of using an ISHS, the participants’ responses were well aligned with the findings of previous studies [29,30,31,93,95,120], indicating that participants considered the perceived benefits factor to outweigh the perceived privacy concerns and comfort. The participants explicitly mentioned that the primary benefit that outweighed the other factors was receiving an effective and rapid response to emergency situations via real-time monitoring. This finding is consistent with that of Wild et al. [31] and confirms those of Qi et al. [95], Reeder et al. [32], and Farina et al. [39], who reported that most participants often suggest the implementation of a feature that enables them to view their activity monitoring in real time. Additionally, the findings from a study by Pal et al. [40] reported that the participants perceived the benefits to outweigh the usability, which was also in accordance with the results obtained in our study. Several participants expressed that they would become accustomed to using the sensors and so, willing to trade-off inconveniences generated in the usability factor for the benefits it provides. Furthermore, all the participants in this study expressed, “*Since benefits of health-related sensors and iAQ sensors are directly related to our well-being, I think it is very important for us*.” implying that the integration of healthcare and iAQ monitoring benefits in a smart home system is needed, and that the integrated benefit will further assist in providing an effective independent living and improving their QoL. 

These findings confirm those of previous studies that identified that elderly participants positively view the acceptance of health-related smart home systems for their potential benefits, which supports safe and independent aging [33,39,115,123]. Although three participants pointed out that the integration of energy consumption monitoring is just as important because of the potential financial benefit it offers, most of the other participants did not feel the significant need for energy consumption monitoring, as they perceived the benefits to be less significant. It is observed from the qualitative analysis that the elderly population perceived the need for energy monitoring to be less significant compared to younger generations who highly consider the importance of saving energy, time, and money, which is further justified in a study by Rasyidah et al. [124]. Moreover, these findings are consistent with those of Parag et al. [34] and Balta-Ozkan et al. [49], which indicated that even though the positively perceived benefits of energy consumption monitoring, the elderly participants question the need to sacrifice their QoL for insignificant financial benefit, which poses the risk of generating negative emotions.

Recommendations for future implementations of smart home systems from previous findings [31,33,120] include providing feedback on sensor monitoring information from professional entities of healthcare and environment management, knowing when to visit doctors [33], smart home systems properly detecting emergency situations [120], and providing effective responses to these immediate needs [31]. These findings were quite similar to the findings obtained in our study: Four participants pointed out that they would like to receive feedback mailed to them from healthcare professionals through the monitoring of their physical activity levels. Three participants pointed out that they suggested receiving information via phone calls only when an anomaly has been monitored, one participant suggested only receiving emergency assistance when detected, and the last remaining participant mentioned that she wanted to be able to view all the collected information personally through the interaction with the ISHS.

Surprisingly, the results obtained from this study are quite different from the many previous findings, for example, Courtney et al. [29] and Hall et al. [94] regarding the non-willingness to actually adopt based on their current need for healthcare benefits. In line with this, other studies by Chung et al. [117], Wild et al. [31], and Coughlin et al. [37] indicated that elderly participants perceive these smart home benefits as only needed for those who would really benefit from it. This can be explained by the fact that they feel that they are still healthy and thus do not require the assistance of smart home sensors; that the elderly participants do not wish to acknowledge their frailty of old age to themselves or to others. Contrary to these findings, all the participants of our study perceived the benefits of the smart home systems to be helpful and thus needed for all the population who employ independent aging-in-place regardless of their age and current health conditions. “*I really hope our country advances more so that in the future, even our children and their children can live in such environment*.” (Participant 5).

Moreover, Coughlin et al. [37] further mentioned that all participants embraced the further development of smart home systems to improve the health and wellness of the elderly population by employing independence through aging-in-place. This was equally consistent in our study, where all the participants expressed that they were very supportive of, and hopeful for further developments of future ISHS to which they were most willing to adopt for a safer and healthier environment. Overall, the primary factor that determined the willingness to adopt an ISHS among elderly participants was the wider range of benefits perceived to outweigh the negative responses of the other factors. However, their willingness was shown only after the participants acquired sufficient awareness of the potential benefits throughout the focus group interview session. The results of this finding are in accordance with Kekade et al. [38], which further indicates that most elderly participants showed willingness after awareness of the benefits of using smart home sensors. Therefore, raising awareness by introducing a wider range of potential benefits that future ISHS aims to provide, the elderly population will be more inclined and willing to practically adopt ISHS.

## 6. Conclusions

This investigation represents the first empirical study to explore elderly users’ perceptions of ISHS through technological trial. Through literature reviews of previous studies, we selected our sensor set based on its accommodation of the benefits that an ISHS provides and implements our ISHS design within nine residential households for our technological trial. Additionally, through the literature, we have explored the implications of the factors derived from the perception of elderly users to determine their willingness to adopt smart home systems. Based on these findings, we identified four popular factors of influence (perceived comfort, perceived usability, perceived privacy, and perceived benefit) that were applied as the guideline criteria for our focus group interview design, which was conducted to investigate the elderly participants’ perceptions of ISHS. From the focus group interviews, we found that the main concerns of the elderly participants pertained to the discomfort of using and wearing smart home sensors, experiencing inconveniences of interruptions to daily activities, and the usability complexity from the application of various smart home sensors, such as the difficulty in controlling sensor functions and understanding the sensor display information.

In particular, stronger negative responses about regularly having to take off smart home sensors to recharge their batteries and putting them back on. One major unexpected finding is that almost all elderly participants positively perceived the ISHS’s invasion of privacy, which was inconsistent with many previous studies [32,37,49,90,117,118]. Rather than generating negative responses from intruding personal privacy, by acknowledging their frailty of old age, the elderly participants emphasized the values of wanting to share their real-time monitoring information with potentially helpful entities, family members, and their friends. This is because they associate their perceived privacy strongly with the benefits of ISHS; it provides them with the feeling of assurance that their health and living conditions for safe independent aging are continuously being monitored. Our findings confirm that elderly users perceive the benefits of ISHS to extend beyond the negative responses generated by the discomfort, usability complexity, and privacy from the application of various smart home sensors, thereby showing a willingness to adopt ISHS.

Nonetheless, although this study investigated elderly users’ perceptions of ISHS, there were several limitations to the generalization of these findings. First, the duration of the technological trial was not conducted long enough to acquire a wider range of the participants’ perception data; the application of longer technological trial duration could provide a wider range of perception data to acquire a better in-depth understanding of the elderly’s responses to their perception of ISHS. For instance, the participants could acquire further perceptions from experiences with various other activities and change their perception from the initial experience of the ISHS. Second, all the participants who volunteered to participate in our technological trial were female, and attitudes were generated differently by gender [34,93]. Moreover, with the sample size of nine participants, all with a South Korean nationality, it is difficult to apply these findings to represent the general perception of the elderly population. Consequently, future investigations with a larger sample size consisting of equal gender ratios and diverse ranges of nationalities could provide a wider scope of elderly perception understanding and thus better represent the elderly population. Lastly, our ISHS sensor-set was selected to best accommodate the essential benefits of ISHS, but to a certain extent, the findings are limited in that the elderly’s perceived benefits and negative responses elicited from the introduced smart home sensors associated with the ISHS sensor set applied in this study. However, it should be noted that ISHS is not limited to being comprised of specific smart home sensors; thus, the integration of various smart home sensors, such as the application of bed sensors and contact sensors, could generate different results.

The contributions of this study include several suggestions regarding future implications for elderly studies on ISHS. For instance, we suggest that future investigators employ non-wearable sleeping sensors that can continuously monitor the physiological levels of the elderly without causing discomfort from interruption to their sleep by wearable smart home sensors. In addition to minimizing discomfort from interruption to daily activities, the placement of visual monitor display sensors should be placed outside the hands reach but are still easily available to see. Moreover, because the integration of various smart home sensors increases the complexity of usability, using simple text to display sensor information and using sensors powered by lightweight replaceable batteries are essential variables that should be taken into consideration, along with providing accessibility to repairs and follow-up services regarding sensor damage. Lastly, for the elderly to practically use ISHS, we emphasize on presenting policies and guidelines regarding terms of privacy to reassure elderly participants about the security of their data and raise awareness of the benefits provided by smart home sensors to elderly users so that they can fully and better understand the potential of using ISHS. The lack of awareness of ISHS benefits and assurance of privacy could lead to different outcomes. The collaboration of these suggestions for future implications can potentially assist in providing a better perceived elderly friendly ISHS environment.

## Data Availability

Data sharing not applicable.

## Appendix A

Predefined guideline questions:

- How do you perceive the comfort in using the sensors?
  1. Did you experience any physical discomfort from wearing the wearable BLE beacon?
  2. Did the design properties of the sensors generate any discomfort? (cues: weight, size, color, and form).
  3. How would you perceive the comfort of wearable sensors at different wearing locations? (cues: head, neck, arm, waist, leg, and ankle)
  4. Did you experience any interruptions to your daily activities from using the sensors?
  5. Do you think changing the installation location of non-wearable sensors/wearing location of the wearable BLE beacon might solve the issues of interruption to daily activities?
- How do you perceive the usability of the sensors?
  1. Did you experience any difficulties interacting (control) with the sensors?
  2. Do you experience any difficulties with the readability of the sensors? (cues: display size, color, and font)
  3. Did you have any worries about damaging the sensors?
  4. Did you experience any difficulties with having to charge the batteries of the sensors?
  5. How would you prefer using replaceable battery cells instead of recharging via charging cable?
  6. Did you experience any issues with forgetting to put the sensor back on after taking it off?
  7. Do you have any suggestions for future implementations regarding the usability of sensors?
- How do you perceive the privacy of the ISHS?
  1. Do you have any privacy concerns regarding sharing information about your activity levels?
  2. What information collected from monitoring do you feel neglected to share? (cues: healthcare, iAQ, and energy information).
  3. To which entities do you feel neglected to share information with? (cues: family, friends, doctors, and caretakers)
  4. If the monitoring information was required to be a long-term collection, how would you feel about it?
- How do you perceive the benefit of the integrated smart home system?
  1. What monitoring information do you think would be most helpful to the elderly population?
  2. How might you like to receive the information? (cues: sensor display, professional feedback, immediate action, when necessary).
  3. What are the benefits of ISHS that you think is most helpful?
  4. Do you think living in an ISHS environment will help improve QoL for the independently aging elderly population?
  5. Would you be willing to adopt ISHS?
  6. Do you have any other suggestions for the future implications of ISHS?
